# Genomic mutation-driven metastatic breast cancer therapy: a single center experience

**DOI:** 10.18632/oncotarget.14476

**Published:** 2017-01-03

**Authors:** Yuan Yuan, Susan E. Yost, Yate-Ching Yuan, Nicola M. Solomon, Isa Mambetsariev, Sumanta Pal, Paul Frankel, Ravi Salgia, Susan L. Neuhausen, Joanne Mortimer

**Affiliations:** ^1^ Department of Medical Oncology & Molecular Therapeutics, City of Hope Comprehensive Cancer Center and Beckman Research Institute, Duarte, CA, USA; ^2^ Bioinformatics Core Facility, City of Hope Comprehensive Cancer Center and Beckman Research Institute, Duarte, CA, USA; ^3^ Department of Biostatistics, City of Hope Comprehensive Cancer Center and Beckman Research Institute, Duarte, CA, USA; ^4^ Department of Population Sciences, City of Hope Comprehensive Cancer Center and Beckman Research Institute, Duarte, CA, USA

**Keywords:** metastatic breast cancer (MBC), genomic profiling, genomic mutation, next-generation sequencing (NGS), targeted therapy

## Abstract

**Background:**

Next-Generation Sequencing (NGS) has made genomic mutation-driven therapy feasible for metastatic breast cancer (MBC) patients. We frequently submit tumor tissue from MBC patients for targeted NGS of tumor using the Illumina HiSeq 2000 platform (FoundationOne®, Foundation Medicine, MA). Herein, we report the results and clinical impact of this test in MBC patients.

**Patients and Methods:**

We identified patients with MBC treated at City of Hope from January 2014 to May 2016 who underwent NGS. Patients’ clinical characteristics, response to treatment (clinical assessment of tumor regression), and genomic mutation profiles were reviewed.

**Results:**

Forty-four patients with MBC underwent NGS: 24 triple negative breast cancer, 16 estrogen receptor positive, and 4 human epidermal growth factor receptor 2 positive patients. Twenty-three patients received more than three lines of chemotherapy prior to NGS. Actionable mutations (potentially responsive to targeted therapies that are on the market or in registered clinical trials) were identified in almost all patients (42/44; 95%) and over half of these 42 patients with actionable mutations (23/42; 55%) initiated mutation-driven targeted therapies. Of these 23 patients, 16/23 (70%) had assessable responses, and 7/23 (30%) were not assessable for response due to short exposure (<2 weeks) or hospice transition. The remaining 19/42 (45%) patients did not initiate targeted therapy.

**Conclusion:**

NGS can identify effective targeted therapy options for MBC patients based on actionable mutations that were not previously offered based on pathology type. NGS should be performed early in patients with good performance status and preferably in clinical settings where genomic mutation-driven therapeutic trials are available.

## INTRODUCTION

Metastatic breast cancer (MBC) is one of the leading causes of cancer mortality in the United States, with over 40,000 deaths annually [1]. Current targeted therapies include everolimus, trastuzumab, lapatinib, and pertuzumab [2, 3]. Identification of actionable targets (those that are potentially responsive to targeted therapy) is urgently needed in patients with MBC who are refractory to standard therapies.

Aberrant mutations are commonly identified in patients with MBC. Screening for targetable genomic mutations and alterations may identify patients who could benefit from specific targeted therapies that may not be typically used in the treatment of breast cancer. As a result of technological advances, high-throughput sequencing, commonly known as “next-generation” sequencing (NGS) is now readily available for clinical use [4]. The success of The Cancer Genome Atlas (TCGA) Project, along with the improved reliability and affordability of NGS, allows the integration of genomic medicine with clinical practice [5].

The concept of using genomic information to guide therapy has become indispensable for precision medicine in cancer treatment. Its feasibility was tested in the SAFIR01/UNICANCER study [6] and in an MD Anderson screening protocol [7]. Both studies concluded that the implementation of genomic mutation/alteration testing is feasible, but only a small percentage of patients with “actionable mutations” were able to enroll onto genotype-matched trials (10.2% and 4.2% in the SAFIR01/UNICANCER and MD Anderson study, respectively) [8].

The concept of genomic-mutation driven therapy is being vigorously tested in the United States. The National Cancer Institute (NCI) recognized the potential of NGS followed by targeted therapy by initiating the Molecular Analysis for Therapy Choice (MATCH) Program. This trial clusters cancers of different types exclusively by genetic mutation and matched targeted therapy. Biopsies from 3000 tumors will undergo NGS to identify individuals whose tumors have genetic abnormalities that may respond to selected targeted drugs. This will be followed by assignment to the genomic-matched phase II study arm [9].

Commercially-available NGS approaches in combination with newer therapeutics targeting genomic mutations has resulted in a paradigm shift to use genomic targeted therapy in routine clinical practice for personalized care of cancer patients. Despite an appreciation for the use of genomics in defining treatment options, genomic mutation-matched clinical trial enrollment remains low [6, 7, 10]. This underscores the value of studying the impact of genomic profiling on treatment of MBC in a real-world setting. At the City of Hope Comprehensive Cancer Center we frequently submit tumor tissue from MBC patients for NGS. Here, we report the results and clinical impact of this test in MBC patients.

## RESULTS

### Patients

We identified 44 patients with MBC treated at City of Hope from January 2014 to May 2016 who underwent NGS; 24 triple negative breast cancer (TNBC), 16 estrogen receptor positive (ER+), and 4 human epidermal growth factor receptor 2 positive (HER2+) patients. Sites of biopsy varied (14 breast, 9 lymph node, 6 skin, 4 liver, 3 lung, 2 bone, 2 brain, one chest wall, one soft tissue, one adrenal gland, and one other). Patient characteristics, treatment history, presence/absence of actionable mutations, response to treatment, and outcomes associated with genomic testing are show in Table 1. The median age at the time of NGS was 54.5 years (range: 34-78). Of the 44 patients, 21 (47%) were non-Hispanic White, 8 (19%) were Hispanic, 7 (16%) were African American, 7 (16%) were Asian, and one (2%) was unknown. Twenty-three patients received more than three lines of chemotherapy prior to NGS.

**Table 1:** Characteristics of patients and outcome associated with NGS

Outcomes		TotalN=44	TNBCN=24	ER+HER2-N=16	HER2+N=4
**Age**	Median (Range)	54.5 (34-78)			
**Race**	Non-Hispanic White	21	8	10	3
	Hispanic	8	7	1	0
	African American	7	6	1	0
	Asian	7	3	3	1
	Other	1	0	1	0
**Lines of therapy prior to FM test**	Median (Range)	3 (0-13)	2 (0-7)	5 (0-13)	5 (2-9)
	0-2 lines	21	15	5	1
	≥ 3 lines	23	9	11	3
**Actionable mutation**	Yes	42	23	15	4
	No	2	1	1	0
**NGS-driven targeted therapy given**	**Subtotal**	**23**	**13**	**9**	**1**
	Therapy duration <2 weeks; transition to palliative care	7	4	3	0
	Therapy duration ≥6 weeks	16	9	6	1
**No NGS- driven targeted therapy**	**Subtotal**	**19**	**10**	**6**	**3**
	Transition to palliative care	7	4	3	1
	Patient's choice	3	3	0	0
	Treated with conventional chemotherapy	5	3	0	1
	Used/exhausted FM recommend therapy prior to FM test	4	0	3	1
**Response to -targeted therapy**	Clinical benefit	8	5	3	0
	Disease Progression	8	4	3	1
	N.A. (could not assess)	7	4	3	0

**Figure 1:**
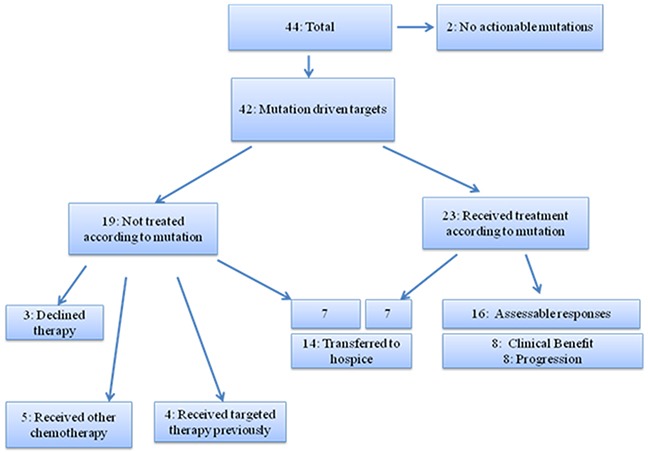
Patient outcome (n=44)

Actionable mutations (potentially responsive to targeted therapies that are on the market or in registered clinical trials) were identified in almost all patients (42/44; 95%) and over half of these 42 patients with actionable mutations (23/42; 55%) initiated mutation-driven targeted therapies (Figure 1). Of these 23 patients, 16/23 (70%) had assessable responses, and 7/23 (30%) were not assessable for response due to short exposure (<2 weeks) or hospice transition. The remaining 19/42 (45%) patients did not initiate targeted therapy: 7 transitioned to palliative care/hospice, 5 received other chemotherapy by treating physicians, 4 exhausted all recommended targeted therapies, and 3 declined treatment (Figure 1). A total of 14 patients (33%) transitioned to palliative care within 2 months of the genomic test result becoming available.

### Treatment response

Among the 24 patients with metastatic TNBC, 13 patients received therapies not traditionally approved for TNBC (e.g. everolimus, pazopanib) and 9 had assessable responses (Table 1). Five patients had clinical benefit assessed by the treating clinician; 4 patients had progression of disease. Four patients could not be assessed for response due to limited drug exposure and/or transition to palliative care. Ten patients were not started on targeted therapy (four transitioned to palliative care; 3 chose not to start targeted therapy; and 3 were currently on other therapy). Of the TNBC group, 4 patients enrolled onto a phase I study, which was available at City of Hope. None of the patients were enrolled onto a genomic mutation-matched clinical trial due to lack of access.

Actionable mutations were identified in 15/16 (94%) patients with ER+/HER2- cancers (Table 1). Nine patients were started on targeted therapy of whom 6 had assessable response (3 clinical benefit, 3 progression of disease), and 3 did not have assessable response due to short exposure of <2 weeks and transition to hospice. Six patients did not receive targeted therapy as 3 had exposure to targeted therapies prior to genomic testing and 3 transitioned to palliative care.

Only one of the 4 patients with HER2+ MBC was treated with targeted therapy based on the NGS result and had progression of disease after enrolling onto two clinical trials. Three patients were not started on targeted therapy; one transitioned to hospice, one exhausted therapy options, and one is currently on other chemotherapy.

### Mutations based on tumor type

**Figure 2:**
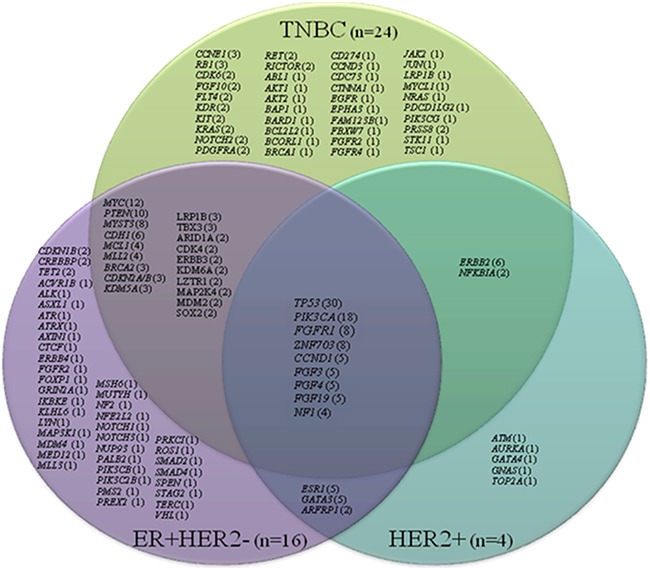
Venn diagram showing number of patients (in parentheses) with specific molecular alterations based on receptor status (TNBC, ER+HER2-, and HER2+) (n=44)

**Figure 3:**
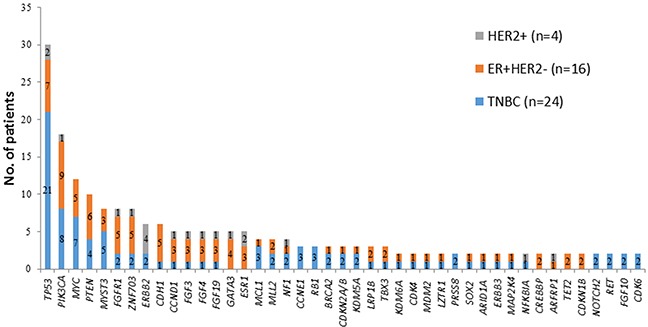
Number of patients with genomic alterations based on receptor status (TNBC, ER+HER2-, and HER2+) (n=44)

**Figure 4:**
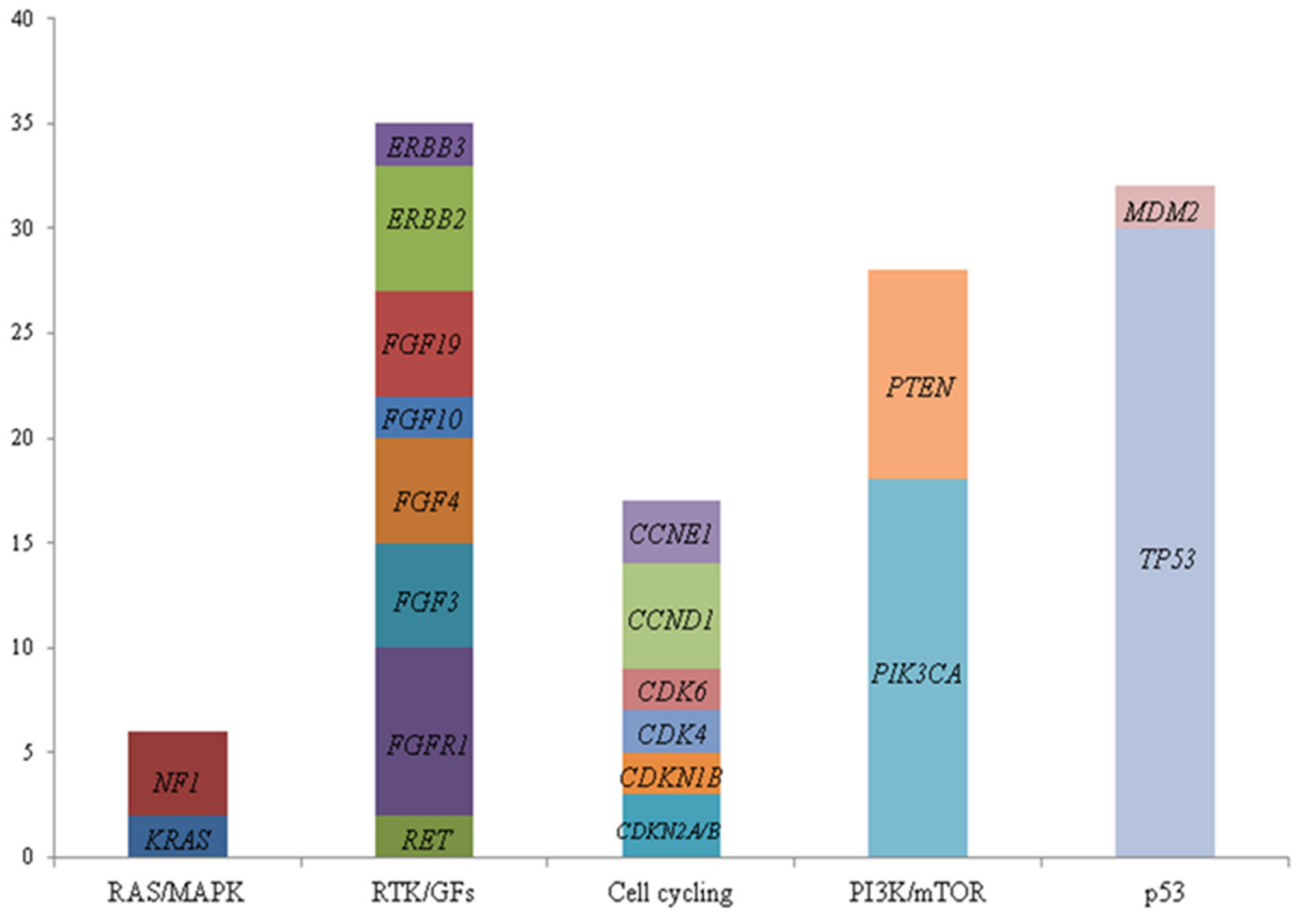
Number of breast cancer patients (n=44) with genetic alterations classified by cell signaling pathways: *RAS/MAPK, RTK/GFs*, cell cycling, *PI3K/mTOR*, and *P53* The ordinate indicates number of patients with alterations. *RAS/MAPK*: Ras GTPase/MAP kinase; *RTK/GFs*: Receptor tyrosine kinase/growth factors; *PI3K/mTOR*: phosphatidylinositol-3-kinase/mammalian target of rapamycin; *P53*: tumor protein p53.

Among three distinctive molecular subtypes, the most common shared alterations identified were *TP53, PIK3CA, FGFR1, ZNF703*, and *CCND1* (Figure 2). *TP53* and *PIK3CA* mutations were the most common genomic alterations observed (Figure 3): *TP53* mutations (n=30; 68%); *PIK3CA* mutation or amplification (n=18; 41%); *MYC* amplification (n =12; 27%); *PTEN* loss (n=10; 23%); *MYST3* (n=8; 18%); *FGFR1* (n=8; 18%); *ZNF703* (n=8; 18%); *ERBB2* mutation or amplification (n=6; 14%); *CDH*1 (n=6; 14%); *CCND1, FGF3, FGF4*, and *FGF 19* (n=5; 11%); *GATA3* (n=5; 11%); and *ESR1* (n=5; 11%). Consistent with other reports, *FGF3*, *FGF4*, and *FGF19* were consistently co-amplified (often with *CCND1*), which can be attributed to the fact that they reside on the same amplicon on chromosome 11 [11, 12]. The number of patients with genetic alterations classified by cell signaling pathways such as *RAS/MAPK, RTK/GFs*, cell cycling, *PI3K/mTOR*, and *p53* are shown in Figure 4.

The genomic mutations and clinical characteristics of the 7 patients who derived clinical benefit are listed in Table 2. The detailed information regarding these genes including function, frequency, and potential therapy, is listed in Table 3. Based on *FGFR1* or *FGFR2* amplifications, 2 patients received pazopanib. One had to stop therapy after 8 weeks due to elevated liver enzymes. Based on mutations in *PIK3CA*, 4 patients received an everolimus-containing regimen. Durable response (>32 weeks) was observed in 3 patients. Interestingly, these 3 patients carry different mutations: *C420R, E545K*-subclonal and *N345K*-subclonal, and *E545K. PIK3CA C420R* is in the tensin-type C2 domain and shows constitutive activation of lipid kinase activity and phosphorylation of *Akt*. *PIK3CA E545K* is a mutation in exon 9 that has shown poor prognosis. *FGFR1* amplification has been correlated with mRNA over-expression, positive ER status, expression of *P53*, and poor prognosis (Table 3).

**Table 2:** Characteristics of the patients with clinical benefit from genomic mutation driven targeted therapy

Pt	Diagnosis	Prior lines of therapy	Mutation/alteration		Therapy(weeks)	Clinical course/Response
1	TNBC	3	***FGFR2 amplification****RET amp-equivocalCCND3 amplificationMCL1 amplification*	*MYC amplificationTP53 G266ECDC73 rearrangement intron 3*	Pazopanib (8)	Therapy discontinued due to elevated liver enzymes/ poor appetite after several dose reductions. Best response/duration: 12 weeks
2	TNBC	6	PIK3CA E726KPIK3CA H1047RNF1 Q1447*CCND1 amplificationBRCA2 E97*FGF19 amplification	*MDM2 amplificationBCL2L2 amp–equivocalCDH1 splice site 531+1G>TFGF3 amplificationFGF4 amplification*	Everolimusplus eribulin trial (8)	Inflammatory breast cancer with chest wall involvement. Response was quick but not durable.
3	TNBC	1	***PIK3CA C420R****CDK6 amplificationMYCL1 amplification*	*TP53 P190delJUN amplification*	Everolimus (34)	Durable response of 34 weeks.
4	TNBC	2	TSC1 L628*TSC1 L628fs*45TP53 T256fs*8	CDKN2A/B lossMLL2 S952fs*38	Everolimus (13)	Significant improvement of systemic disease for 12 weeks. Then brain metastases and transition to hospice after whole brain radiation therapy.
5	ER+HER2-	2	***PIK3CA E545K-subclonalPIK3CA N345K-subclonal***FGFR1 amplificationARID1A G87fs*24	ARID1A R356fs*44CDH1 D433NZNF703 amplification	Everolimusplus exemestane (24)	Progression on letrozole plus palbociclib prior switching to exemestane plus everolimus. Still on treatment after 32 weeks
6	ER+HER2-	5	***FGFR1 amplification***MYC amplificationCREBBP Q943*	*MYST3 amplificationZNF703 amplification*	Pazopanib (32)	Duration of clinical benefit: 8 months on pazopanib with SD. 7/22/14 – 3/16/2015 –mastectomy. Post-op endocrine therapy till 8/21/15 liposomal doxorubicin till 9/14/15. Then eribulin 10/12/15 till now
7	ER+HER2-	2	***PIK3CA E545K***MAP2K4 T78fs*10	Everolimusplus exemestane (32)	Progressed after durable response of 32 weeks	

**Bold**: Actionable mutations.

**Table 3:** Actionable mutations and genomic mutation driven targeted therapy†

Mutations	Gene Function	Frequency in breastcancer and prognosis	Potential treatment
***FGFR1* amplification** Fibroblast growth factor receptor 1 (Fgfr1)	Cell cycle and angiogenesis; upstream regulator of *Ras, MAPK*, and *Akt* signaling pathways	10-17%; poor prognosis	*FGFR* family inhibitors (pazopanib, regorafenib, ponatinib). Overexpression may be a mechanism of acquired resistance to gefitinib. *FGFR1* amplification may contribute to resistance to hormonal therapy in ER+ breast cancers
***FGFR2* amplification** Fibroblast growth factor receptor 2 (Fgfr2)	Tyrosine kinase cell surface receptor (cell differentiation, growth, angiogenesis)	1-11.5%; associated with resistance to chemotherapy	F*GFR* family of inhibitors (pazopanib, regorafenib, ponatinib)
***PIK3CA E726K, PIK3CA H1047R*** Protein p110-alpha, catalytic subunit of *PI3K*	Cell signaling regulating cell growth, proliferation, differentiation, motility, and survival	25-40%; *H1047R* associated with better prognosis than *E542K, E545K* mutations	*PI3k/AKT/mTOR* inhibitors (everolimus and temsirolimus); other *mTO*R inhibitors; *PI3K* and *Akt* inhibitors alone or in combination; *PI3K* alpha-specific *BYL719. CDK4/6* inhibitors may sensitize *PIK3CA* mutant breast cancer to *PI3K* inhibitors.
***PIK3CA C420R*** Protein p110-alpha, catalytic subunit of *PI3K*	Cell signaling regulating cell growth, proliferation, differentiation, motility, and survival	26-38.5%	*PI3k/AKT/mTOR* inhibitors (everolimus and temsirolimus); other *mTOR* inhibitors; *PI3K* and *Akt* inhibitors alone or in combination; *PI3K* alpha-specific BYL719 *CDK4/6* inhibitors may sensitize *PIK3CA* mutant breast cancer to *PI3K* inhibitors.
***PIK3CA E545K* – subclonal *PIK3CA N345K* – subclonal** Protein p110-alpha, catalytic subunit of *PI3K*	Cell signaling regulating cell growth, proliferation, differentiation, motility, and survival.	25-40%	*PI3k/AKT/mTOR* inhibitors (everolimus and temsirolimus); other *mTOR* inhibitors; *PI3K* and *Akt* inhibitors alone or in combination; *PI3K* alpha-specific BYL719 *CDK4/6* inhibitors may sensitize *PIK3CA* mutant breast cancer to *PI3K* inhibitors; combined HER2+ *PI3K* pathways may be necessary in tumors with *ERBB2* amplification and *PIK3CA* mutation.
TSC1 L628*, TSC1 L628fs*45 Protein hamartin	*TSC1* forms heterodimer with *TSC2* that acts as a GTPase activating protein for Rheb, a potent activator of the mammalian target of rapamycin (*mTOR*).	<1%	*mTOR* inhibitors (everolimus, temsirolimus, exemestane). Loss of *TSC1* leads to activation of *mTOR* and therefore may predict sensitivity to *mTOR* inhibitors.

†Information provided through Foundation Medicine®

## DISCUSSION

In this single-center, retrospective analysis, we have shown that a genomic mutation profiling-based approach is feasible in identifying targetable genomic alterations and mutations in patients with advanced MBC. Genomic profiling led to treatment with molecular targeted therapy in 23 of 44 patients (52%). Of those, 7 had clinical response, highlighting the potential utility of the genomic profiling tool in this population. Unfortunately, 14 of 42 (33%) patients deteriorated quickly prior to initiation of targeted therapy or had a very short exposure to the targeted therapy (<2 weeks). None of the patients were enrolled onto matched genomic mutation-targeted therapeutic trials due to lack of access. The most commonly prescribed targeted therapy in this study was everolimus, which largely reflects the prevalence of phosphatidylinositol 3-kinase/mammalian targets of rapamycin (*PI3K/mTOR)* pathway alterations in the breast cancer population. This is a major pathway involved in the regulation of cell survival and proliferation, and it is the most frequently altered pathway in breast cancer [13]. Despite the encouraging findings, few other genomic mutations were targeted due to lack of access. These findings suggest that genomic testing must be offered early in the course of MBC treatment to allow access to targeted therapy and the opportunity to assess response. In addition, access to genomic mutation-driven clinical trials is most critical in utilizing genomic driven therapy. This finding is supported by other studies, and adds to the body of literature applying genomic medicine to real-world practice in breast cancer.

Lack of access to genotype-matched targeted therapy trials is a recurring theme across several studies. Parker *et al*. reported on the utilization of a multidisciplinary molecular tumor board in optimizing the management of 43 patients with advanced heavily-pretreated breast cancer undergoing genomic testing [11]. Seventeen of the 43 patients (40%) were treated with targeted therapy; 7 (16%) had stable disease for ≥6 months (n=2) or partial remission (n=5) [11]. Lack of access to targeted therapy was the main reason that patients could not be treated.

Based on recent large center experiences, genomic mutation-driven therapy has not yet been shown to improve patient's quality of life or clinical outcome. Recently Meric-Bernstam *et al*. reported a genomic mutation screening study of 2000 patients with advanced cancer who underwent a genomic testing protocol. Thirty nine percent of patients had ≥1 actionable mutation, but only 11% (n=83) of those were enrolled onto genotype-matched clinical trials. This translates to 4.1% of total patients screened [14]. Similarly, the SAFIR01/UNICANCER trial was designed as a multi-center molecular screening study to identify genomic mutations/alterations in breast cancer patients to provide matched targeted therapy [6]. Of 423 patients enrolled, genomic analyses led to potential matched targeted therapy in only 55 (13%) patients due to limited availability of therapeutic agents. Forty three (10%) patients received targeted therapy, 4 had an objective response, and 9 had stable disease for over 4 months [6]. Collectively, these studies underscore the urgent need to discover more effective molecularly targeted agents, refine treatment algorithms to take drug combinations into account, and investigate this approach at an earlier clinical time point.

Mutation profiles of refractory breast cancers in the current study were heterogeneous and none of the tumors carried the same genomic mutation profile; this is consistent with previous studies [15, 16]. Of the mutations identified, *TP53* loss and*PIK3CA* mutation were the most common genomic alterations observed in this cohort of patients. Hyperactivation of the *PI3K* pathway occurs in 70% of breast cancers; and approximately 30% of breast cancers have mutations in *PIK3CA* [13]. *PIK3CA* mutation in TNBC has been reported with variable frequency, from 13% to 23.7% across several studies [8, 17–19]. Comparing the genomic mutation profiling with TCGA database, which mainly analyzed primary TNBC at the time of initial surgery, our heavily pretreated TNBC tumors carried a higher percentage of *PIK3CA* mutations (29% vs. 8%, p<0.01). In contrast, there were only moderate changes identified in the ER+HER2- population comparing metastatic/resistant tumors with primary tumors in the TCGA database. It is suspected that in refractory metastatic tumors, the mutation burden of this pathway may increase due to selection pressure from chemotherapies. The significance of these findings remains to be determined but may be attributed to small sample size and tumor heterogeneity.

Increasing evidence has demonstrated that the mutational landscape of tumors can change upon treatment with chemotherapy and/or targeted therapies with both gain and loss of actionable alterations. When alterations not detected in the original biopsy are present in a subsequent biopsy, it remains unclear whether these represent new mutations or selection for rare sub-clones already present in the primary tumor [7]. In order to understand individual tumor evolution with treatment selection pressure, we are currently conducting a paired tumor tissue genomic analysis study comparing primary and recurrent/refractory TNBCs. We will test if targeted exome sequencing can capture the heterogeneity in a tumor and the sub-clones that contribute to therapy resistance.

Our study is limited by small sample size and retrospective approach. This study used a targeted exome sequencing method without normal breast tissue control. A recent study by Jones *et al*. emphasized the importance of paired tumor-normal tissue analysis for precise identification and interpretation of somatic and germline alterations, which may impact management of patients [20]. In addition, genomic mutation-driven targeted therapies were recommended by each individual treating physician. Ideally, a molecular tumor board discussion with consensus agreement should be implemented for this approach. Additionally, during the study period, there were no genomic mutation-driven clinical trial options available. Lastly, one third of patients could not be treated or evaluated because of transition to palliative care or hospice, which limited the accessibility of the targeted agents in the metastatic setting.

## METHODS

### Patients

A team of physicians identified patients with MBC treated at City of Hope from January 2014 to May 2016. Archival tumor samples obtained from standard diagnostic or therapeutic procedures were tested with Clinical Laboratory Improvement Amendment-certified targeted NGS (FoundationOne®, Foundation Medicine, MA) using the Illumina HiSeq 2000 platform. FoundationOne® is a comprehensive genomic profile that applies NGS to identify base substitutions, insertions and deletions (indels), copy number alterations (CNAs), and rearrangements using formalin-fixed paraffin-embedded (FFPE) samples. FoundationOne® applies NGS across all genes known to be unambiguous drivers of solid tumors with high accuracy by sequencing the coding regions of 315 cancer-related genes plus introns from 28 genes often rearranged or altered in cancer to a typical median depth of coverage of greater than 500x. Eligible patients had stage IV breast cancer requiring systemic therapy. Patients’ age, demographics, tumor pathology types, stage and treatment histories, response to treatment, and genomic mutation profiles were collected by electronic medical chart review. FoundationOne® reports were reviewed.

### Assessment of treatment response

The treatment response was based on retrospective review of restaging imaging using x-ray computerized tomography (CT) and bone scans, and/or treating physician's clinical assessment (not response evaluation criteria in solid tumor (RECIST) criteria).

### Analysis of genomic mutation data

We generated a database of all genomic alterations based on the FoundationOne® reports. Descriptive statistics were used for this study.

## CONCLUSION

Targeted genomic sequencing tools such as NGS can identify alterations that may respond to targeted therapies that have not generally been used based on pathology type alone. NGS should be performed early in patients with good performance status. We predict an increased use of the test in the community, although we recognize this approach should ideally be utilized in a setting where genomic mutation-driven therapeutic trials are available. In addition, customized combinations of targeted therapy, along with novel clinical trial design, may be needed to make genomic mutation-driven cancer medicine feasible in order to overcome tumor heterogeneity.

## Abbreviations

ER+: Estrogen receptor positive
HER2+: Human epidermal growth factor receptor 2 positive
MATCH: Molecular Analysis for Therapy Choice
MBC: Metastatic breast cancer
NCI: National Cancer Institute
NGS: Next-Generation Sequencing
TCGA: The Cancer Genome Atlas
TNBC: Triple negative breast cancer
