# Correction: Genomic mutation-driven metastatic breast cancer therapy: a single center experience

**DOI:** 10.18632/oncotarget.20632

**Published:** 2017-09-05

**Authors:** Yuan Yuan, Susan E. Yost, John Yim, Yate-Ching Yuan, Nicola M. Solomon, Isa Mambetsariev, Sumanta Pal, Paul Frankel, Ravi Salgia, Susan L. Neuhausen, Joanne Mortimer

**Affiliations:** ^1^ Department of Medical Oncology & Molecular Therapeutics, City of Hope Comprehensive Cancer Center and Beckman Research Institute, Duarte, CA, USA; ^2^ Bioinformatics Core Facility, City of Hope Comprehensive Cancer Center and Beckman Research Institute, Duarte, CA, USA; ^3^ Department of Surgery, City of Hope Comprehensive Cancer Center and Beckman Research Institute, Duarte, CA, USA; ^4^ Department of Biostatistics, City of Hope Comprehensive Cancer Center and Beckman Research Institute, Duarte, CA, USA; ^5^ Department of Population Sciences, City of Hope Comprehensive Cancer Center and Beckman Research Institute, Duarte, CA, USA

**This article has been corrected:** Dr. John Yim was added to the author list.

The authors sincerely apologize for this oversight.

Original article: Oncotarget. 2017; 8:26414-26423. https://doi.org/10.18632/oncotarget.14476

